# Delirium followed by neuroleptic malignant syndrome in rehabilitation setting. Is it anger reaction before discharge?

**DOI:** 10.11604/pamj.2013.15.26.2218

**Published:** 2013-05-18

**Authors:** Rafat M Al Owesie, Asirvatham Alwin Robert

**Affiliations:** 1 Department of Psychiatry and Psychology, Medical Affairs, Sultan Bin Abdulaziz Humanitarian City, Riyadh, Saudi Arabia; 2 Research Center, Medical Affairs, Sultan Bin Abdulaziz Humanitarian City, Riyadh, Saudi Arabia

**Keywords:** Neuroleptic Malignant Syndrome, antipsychotics, Saudi Arabia

## Abstract

Delirium and neuroleptic malignant syndrome (NMS) are two uncommon syndromes that are often unrecognized or misdiagnosed by the primary physicians as functional psychiatric disorders. The infrequency and the heterogeneity of clinical manifestation, progression and outcome with which those diagnoses are encountered (particularly in chronic rehabilitation settings) make them formidable diagnostic challenge. In this report, we present a case in which there was a misdiagnosis of delirium and NMS as functional psychiatric disorders. This report demonstrates the fact that early recognition and intervention in such cases are essential to prevent the grave outcome and potential fatality. The authors believe that there is a key role for the consulting psychiatrist in this area in providing diagnostic clarity and advice regarding management and ongoing staff education.

## Introduction

Neuroleptic malignant syndrome (NMS) is an idiosyncratic reaction usually due to the effects of neuroleptic drugs, characterized by hyperthermia, muscle rigidity, mental status changes, motor abnormalities (bradykinesia and muscle rigidity), autonomic dysfunction (blood pressure instability, diaphoresis and tachycardia), hyperthermia and altered consciousness [1–6]. Several laboratory abnormalities such as elevated serum levels of creatine phosphokinase (CPK), aldolase, transaminases, lactic acid dehydrogenase, decreased serum iron concentrations, metabolic acidosis and leukocytosis are associated with NMS, though none are specific for diagnosis [2, 4–8]. Although potent neuroleptics are more often related with NMS, all antipsychotic agents, typical or atypical, may precipitate the syndrome. NMS has also been linked with nonneuroleptic agents that block central dopamine pathways (e.g., metoclopramide, amoxapine, and lithium) [8–10].

The incidence of NMS once ran is estimated as high as 3% of patients treated with antipsychotics (AP), more recent data suggest an incidence of 0.01% - 0.02% [10]. This decrease in incidence reveals the improved alertness of the NMS, more conservative prescribing patterns, and the shift to use of atypical antipsychotics. Despite its decreasing incidence, NMS remains an important cause of morbidity and mortality among patients receiving antipsychotics [5, 9].

Diagnosis and treatment of NMS can be difficult and the syndrome complicates further psychiatric treatment. Absence of immediate and appropriate therapy may result in, mortality and in some cases NMS can only be diagnosed after death of a patient [5, 11]. Treatment is mainly supportive which includes withdrawal of AP or other causative agent, and occasional use of drugs such as dopaminergic agonist (bromocriptine) and dantrolene [3, 5].

In this study we report a 30-year-old Saudi gentleman with spinal cord and mild traumatic brain injuries who developed neuroleptic malignant syndrome (NMS) shortly after receiving two doses of intramuscular antipsychotics given to control agitation due to delirium. This patient had atypical NMS presentation and needed intensive care unit (ICU) admission. The hallmark of the presentation was elevated CPK.

## Patient and observation

A 30-year-old Saudi gentleman who was admitted to the hospital for rehabilitation. Patient had a motor vehicle accident one month before admission which resulted in multiple injuries (including spinal cord injury and mild traumatic brain injury). He did not manifest behavioral or psychological problems during the initial four months stay at the hospital. Patient was supposed to be discharged from the hospital after achieving the rehabilitation goals.

### Neuroleptic malignant syndrome and patient behavior

In the beginning of the fourth month, the patient was referred to our psychiatric services from the spinal cord injury rehabilitation unit for evaluation and management of agitated combative behavior, he started to manifest abruptly one day after he was informed of imminent discharge. Physician in the ward, thought that this frustrated angry behavior was due to patient's dissatisfaction of rehabilitation achievements. Detailed review found no prior psychiatric history, history of substance abuse and known exposure to neuroleptics. Patient had one previous episode of urinary tract infections (UTI) during his hospital stay that required treatment with oral antibiotics.

On the first day of assessment, the patient's initial signs and symptoms included a temperature of 38.4°C, agitated and combative behavior, refusing medication (including muscle relaxants) and oral intake and progressively deteriorating sleep. The provisional diagnosis was delirium due to a recurrent UTI. Olanzapine 10 mg IM stat was given and urinalysis (UA) was ordered. On the following day, (UA) revealed evidence of urinary tract bacterial infection for which an intravenous antibiotic was commenced. On the same day the patient continued to have agitated combative behavior for which he received haloperidol 10 mg IM in the afternoon (which calmed him down).

On the third day the patient demonstrated a clear change in his mental state. His agitation decreased, however he began to show perplexed affect, distractibility and poor initiation of purposeful behavior. He was immobile and unresponsive, staring and not responding to verbal commands but responding to pain with purposeful withdrawal. Assessment of central nervous system (CNS) was difficult as the patient was constantly moving and had generalized spasticity. He responded to questions by uttering a few words and became very agitated, shouting obscenities.

Laboratory results done on the same day showed the following results: white blood cell count: 18.2 cells/ml, serum creatinine: 93 µmol/L, fasting blood glucose: 8.37 mmol/l, calcium:2.59 mmol/l, phosphorus: 1.79 mmol/l, alkaline phosphatase (ALP):162 IU/L, aspartate aminotransferase (AST):76 U/L, gamma-glutamyltranspeptidase (GGT): 157 U/L, and creatinine phosphokinase (CPK) level: 4731 IU/L. The urine output was low at 350ml/12h.

The patient was started on hydration and immediately transferred to ICU with differential diagnoses that included NMS and meningitis. Early the next morning the patient had a complex partial seizure which ceased after IV diazepam administration. Lumbar puncture (LP), computerized tomography (CT) of the head and electroencephalogram (EEG) did not indicate any significant findings. The diagnosis of NMS was retained, and the patient was started on bromocriptine 7.5 mg three times daily and lorazepam 1 mg at bedtime.

**Figure 1:**
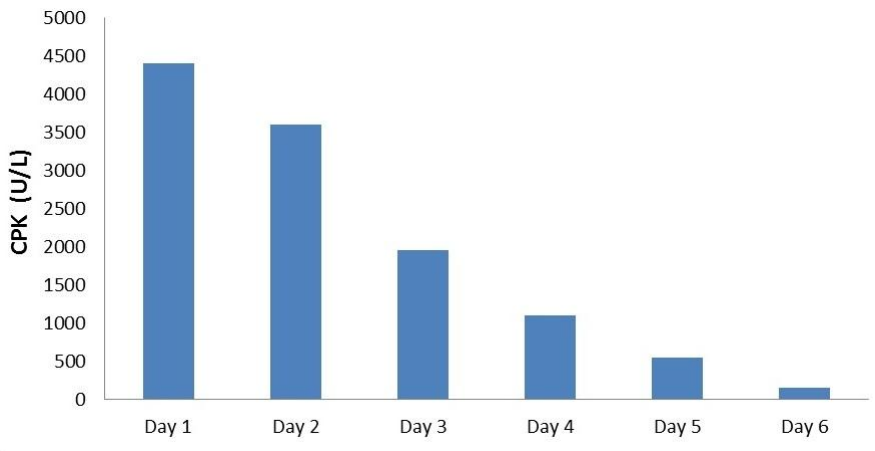
Creatinine phosphokinase (CPK) level by day

On the 5th day after the (ICU) admission the patient demonstrated decreased rigidity and agitation and increased periods of wakefulness. On the 6th day patient regained a full level of orientation and cooperation. Liver function tests and CPK normalized (Figure 1) and patient was transferred to the general ward.

## Discussion

Physicians should have a high index of suspicion of atypical NMS particularly in non-psychiatric patients who present with a change in mental state or fever after getting an antipsychotic. This case was unusual because of the rapid development of NMS after two doses of intramuscular antipsychotics and a maximal temperature of only 38.4°C. In addition, there was a great difficulty in assessing rigidity because of quadriparesis and generalized spasticity the patient has as a consequence of traumatic spinal cord and brain injuries. Studies reported many pharmacological and treatment variables that could be considered as risk factors for development of NMS. Nearly all dopamine antagonists are associated with NMS, although high potency conventional antipsychotics are associated with a greater risk when compared to low-potency agents and atypical antipsychotics [1, 4–5, 8, 10].

Several clinical, systemic, and metabolic factors have been correlated with the incidence of NMS, including agitation, dehydration, poor oral intake, restraint, preexisting abnormalities of CNS dopamine activity or receptor function, iron deficiency, traumatic brain injury, sudden stopping of muscle relaxant (dantrolene) and psychological stress of physical disease [5, 12–13]. It is obvious that our patient had many of the above mentioned risk factors that predisposed him to develop NMS.

A number of laboratory abnormalities such as elevation of serum liver enzymes and leukocytosis are connected with NMS, while none are specific for the diagnosis [2, 4–8]. In this case we found abnormalities in white blood cell count 18.2 cells/mL, serum creatinine 93 µmol/L, fasting blood glucose 8.37 mmol/L, calcium 2.59 mmol/l, phosphorus 1.79 mmol/l, alkaline phosphatase (ALP) 162 IU/L, aspartate aminotransferase (AST) 76 U/L and gamma-glutamyl transpeptidase (GGT) 157 U/L. CPK elevation is one of the most reliable indicators of NMS. In NMS, CPK is typically more than 1000 U/L and can be as high as 100,000 [13–14]. The degree of CKP elevation correlates with disease severity and prognosis. Normal CKP can be seen if rigidity is not well developed, particularly during early onset of the syndrome. In our case, there was a moderate elevation and a rapid decrease of CPK level over the course of 6 days.

The treatment for NMS should be individualized and empirically based on the character, duration, and severity or stage of clinical signs and symptoms 5. Intensive medical care should include careful monitoring for complications such as cardiorespiratory failure, renal failure, aspiration pneumonia, and coagulopathies. The care may involve support of cardiac, respiratory, and renal function. For mild cases, supportive care and careful clinical monitoring may be sufficient [5], whereas in severe cases, all antipsychotic medications should be stopped immediately; cooling measures and aggressive medical management, including intravenous fluids should be initiated in an intensive care setting. Benzodiazepines particularly lorazepam have been recommended as first-line intervention in patients with NMS [15]. Dopaminergic agents particularly bromocriptine may reverse Parkinsonism in NMS and have been reported in case reports and meta-analyses [5, 15].

## Conclusion

This report demonstrates the fact that early recognition and intervention in such cases are essential to prevent the grave outcome and potential fatality. The authors believe that there is a key role for the consulting psychiatrist in this area in providing diagnostic clarity and advice regarding management NMS. Educating health care professionals, patients and even caregivers about this uncommon condition increases the chance of early diagnosis, immediate intervention and reduces the risk of mortality.
